# Neurosurgical Complications Following Tooth Extraction: A Systematic Review and Individual Patient Meta-Analysis: Neurosurgical Complications Following Tooth Extraction

**DOI:** 10.31661/gmj.v13iSP1.3570

**Published:** 2024-12-31

**Authors:** Shahram Shafa, Elaheh Entezar-Almahdi, Amir Hossein Pourdavood, Behzad Vosooghinezhad, Mohammad Zarenezhad, Armin Jodaei, Narges Ghafari, Lohrasb Taheri, Tayyebeh Zarei, Nastaran Bagheri, Mojtaba Ghaedi, Marjan Kazemi Nia, Mansoor Deilami

**Affiliations:** ^1^ Department of Orthopedics, Peymanieh Hospital, Jahrom University of Medical Sciences, Jahrom, Iran; ^2^ Assistant Professor of Pharmaceutics, Jahrom University of Medical Sciences, Jahrom, Iran; ^3^ Department of Surgery, Peymanieh Hospital, Jahrom University of Medical Sciences, Jahrom, Iran; ^4^ European University, Tbilisi, Georgia.; ^5^ Legal Medicine Research Center, Legal Medicine Organization, Tehran, Iran; ^6^ Critical Care and Pain Management Research Center, Hormozgan University of Medical Sciences, Bandar Abbas, Iran; ^7^ Department of Oral and Maxillofacial Radiology, Golestan University of Medical Sciences, Gorgan, Iran; ^8^ Department of Anesthesiology and Critical Care, School of Medicine, 5th Azar Hospital, Sayyad Shirazi Hospital, Golestan University of Medical Sciences, Gorgan, Iran

**Keywords:** Neurosurgical Complications, Tooth Extraction, Neurosurgical Procedures, Brain Abscess; Meningitis, Cerebrovascular Accidents, Systematic Review and Meta-Analysis

## Abstract

**Background:**

We aimed to review the characteristics of patients with neurosurgical complications after tooth extraction.

**Materials and Methods:**

This systematic review followed PRISMA guidelines and searched PubMed/MEDLINE, Embase, Web of Science, and Scopus databases for studies investigating neurosurgical complications post-tooth extraction. Relevant keywords for dental extraction, adverse events or complications, and neurosurgery were searched using Boolean operators. Extracted data was synthesized using proper statistical tests.

**Results:**

Among 42 studies, 47 cases (34 males, 13 females) were included. The complications were distributed as follows: 25 brain abscesses, 11 meningitis cases, 8 cerebrovascular accidents, 2 cases with both meningitis and stroke, and 1 pituitary macroadenoma. Four deaths occurred in cerebrovascular accident cases. A significant association was found between preexisting diseases and death (odds ratio = 2.15, 95% CI: 1.08-4.29, P-value = 0.03). Three mucormycosis and two mycobacterium tuberculosis cases were reported. The most common symptoms were headache (55.32%), fever (38.3%), and laterality symptoms (25.53%). Neck pain/neck rigidity was more prevalent in females (30.77% vs. 8.82%, P = 0.042), as were nausea and vomiting (30.77% vs. 8.82%, P = 0.028). Overall, 31.91% of cases had no underlying diseases. The mean time from tooth extraction to emergency room visit was 19.73 days (SD = 31.01 days), ranging from 2 to 180 days. Fourteen cases (29.79%) involved the upper jaw, 6 (12.77%) the lower jaw, and 2 (4.26%) both jaws.

**Conclusion:**

The study introduces a novel approach by systematically reviewing and analyzing individual patient data to identify specific risk factors and symptoms associated with neurosurgical complications following tooth extraction. Healthcare providers can use the identified symptoms, such as headache and fever, as key indicators for prompt evaluation and management of patients presenting after tooth extraction, especially in male patients with pre-existing conditions who are undergoing upper jaw teeth extraction.

## Introduction

Interaction between dental sciences and neurosurgery is multifaceted, as is the shared anatomy of the head and neck region [1], the emergence of the concept of the "brain-oral axis" in neurosciences [2], and the effect of dental health on neurological diseases [3]. Collaborations between dentists and neurosurgeons in combined surgeries have become increasingly common, like combined craniomaxillofacial and neurosurgical procedures [4].

Dental caries and its complications are significant reasons for tooth extractions, with a high prevalence observed in the population [5]. Socio-demographic factors like gender may also influence extraction rates, with higher prevalence observed among female individuals [6]. Dental extractions, while common procedures, can lead to adverse events, such as pain and discomfort, swelling and bruising, bleeding, infection, and nerve damage [7][8]. Inferior Alveolar Nerve (IAN) is susceptible to injury during mandibular tooth extractions [9][10]. Lingual nerve and trigeminal nerve injuries might also happen [9][10]. Pneumomediastinum, pneumorrhachis, pneumothorax, and pneumopericardium, are infrequently encountered but documented [11]. Another uncommon complication is surgical emphysema [12], typically associated with the use of high-speed air rotors during extractions. Additionally, osteoradionecrosis, characterized by bone tissue death due to prior radiation therapy, and complications like bite collapse and improper tooth alignment can occur post-extraction, albeit rarely [13]. Severe trismus, although not exceedingly common, may also manifest after extractions, leading to difficulties in mouth opening [14]. Neurosurgery following tooth extraction is a rare occurrence but may be necessary in cases where dental procedures inadvertently lead to complications involving nearby neurological structures. Traumatic neuralgia and posttraumatic pain syndrome have been reported as complications necessitating neurosurgical evaluation after dental procedures [15]. In some cases, neurosurgery may be necessary to decompress the inferior alveolar nerve after endodontic treatment complications [16]. Additionally, microsurgical repair of lingual nerve injuries may be required in cases of nerve damage during third molar removal [17]. A review focused on the neurological complications associated with local anesthesia in dentistry, including adverse effects such as diplopia, ptosis, ocular paralysis, blindness, paresthesia, trismus, neuralgia, and facial palsy [18]. Another review investigated the neurological sequelae following surgical interventions on the lower molars, including adverse effects such as transient and permanent sensory deficits, often resulting from the compression or irritation of the mandibular nerve [19].

As the existing literature on neurosurgical complications following tooth extraction is fragmented and lacks a comprehensive analysis of individual patient data, we aimed to systematically review and meta-analyze the characteristics of patients with neurosurgical complications after tooth extraction. Furthermore, previous studies have primarily focused on specific aspects of dental procedures or local anesthesia, without providing a thorough understanding of the risk factors and symptoms associated with neurosurgical complications. Our study introduces a novel approach by synthesizing individual patient data to identify specific risk factors and symptoms that can serve as key indicators for prompt evaluation and management of patients presenting after tooth extraction.

## Materials and Methods

A systematic review was conducted to investigate neurosurgical complications following tooth extraction. The Preferred Reporting Items for Systematic Reviews and Meta-Analyses (PRISMA) guidelines were followed for reporting the findings of this systematic review [20].

### Information sources and Search strategy:

A search strategy was developed using relevant keywords and Medical Subject Headings (MeSH) terms. The following databases were searched from inception to January 2024: PubMed/MEDLINE, Embase, Web of Science, and Scopus. The search strategy utilized the following combination of terms:

("dental extraction" or "tooth extraction" OR "milk tooth" OR "dental Manipulation" OR "dental extraction" ) AND ((brain) OR (neurosurgery) OR (stroke) OR (spine) OR (cerebrovascular event)

### Eligibility criteria

Inclusion criteria: Studies that investigated neurosurgical complications associated with dental extraction procedures. Published in peer-reviewed journals and available in English language were included. Studies with individual patient data were only included. To diagnose neurosurgery complications originating from dental sources, three conditions should be met: the absence of alternative bacteremia sources, a microbiological profile in line with oral flora, and clinical or radiographic indications of dental infection [21].

Exclusion criteria: Orbital abscess cases were excluded. Sinusitis-related infections were not included. Iatrogenic traumatic brain injury cases were not included. Cluster Headache cases were not counted as neurosurgical cases.

### Selection and Data Collection Process

Two independent reviewers screened titles and abstracts of retrieved articles based on the predefined inclusion and exclusion criteria. Full texts of potentially relevant articles were then assessed for eligibility. Any disagreements between the reviewers were resolved through discussion or consultation with a third reviewer. A standardized data extraction form was used to extract relevant information from included studies. Data extracted included study characteristics (author, year of publication, study design), participant demographics, details of dental extraction procedures, neurosurgical complications reported, and relevant outcomes.

### Study risk of bias assessment

The methodological quality of the included studies was evaluated using The CARE guideline of case reporting [22].

### Synthesis methods

Data synthesis was performed summarizing findings from included studies, including the prevalence and types of neurosurgical complications following dental extraction. Meta-analysis was performed using STATA software, with descriptive statistics of n (%) for categorical data and mean±SD for continuous ones. Chi-square and independent t-tests were used to test various hypotheses, considering the significant value lower than 0.05.

## Results

**Figure-1:**
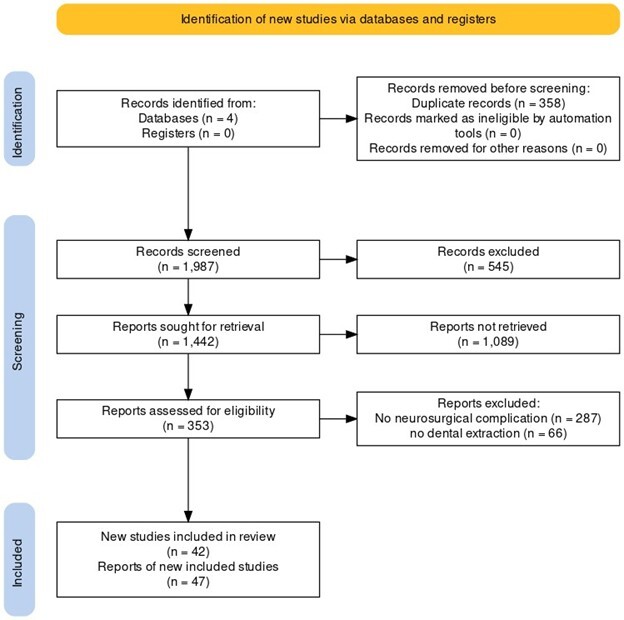
Figure 1. PRISMA flowchart of study showing steps taken from edibility assessment to study selection

**Table:** Table1. Characteristics of included studies

	**Age**	**Gender**	**Comorbidities**	**Symptoms on ER Admission **	**Extracted Tooth (Tooth Identity) **	**CSF culture organism **	**Final Diagnosis **
Amorim *et al*.	67	female	none	headache, neck pain, no fever	Upper left second molar	ceftriaxone-sensitive Streptococcus intermedius	OBA with hydrocephalus
Kroppenstedt et al.	69	male	hypothyroidism	Headache, dizziness, fatigue, blood pressure variations, left thoracic pain	Three teeth in the lower left jaw	none	Pituitary macroadenoma
Calderon-Miranda	26	female	missing	Headache, nausea, emesis	missing	missing	Subdural hematoma
Pallesen et al.	55	male	none	Acute onset of weakness in the left leg	Professional tooth cleaning	Streptococcus intermedius and Staphylococcus warneri	Multiple BAs; subsequent complications included subdural empyema and focal epileptic seizures
Hollin et al. (a)	19	male	missing	Headaches, greenish foul discharge from the right nostril, fever (2 days)	Two carious right upper molar teeth extracted	Staphylococcus CJWXU.S, nonhemolytic streptococcus	subdural empyema and diffuse leptomeningitis
Hollin et al. (b)	31	female	missing	Headaches, painful swelling in the right jaw	Right lower molar	sterile	Subdural empyema
Hollin et al. (c)	36	male	none	Personality changes, Dysarthria, focal convulsions, weakness, numbness of the right side, insomnia, lethargy, generalized malaise, fever	A right upper premolar tooth	missing	parietal abscess
Hollin & Gross (a)	25	male	missing	Headaches, malaise, drowsiness, confusion	Fourteen upper teeth	missing	Subdural empyema
Hollin & Gross (b)	38	male	missing	Headaches, fever, mental changes	Infected tooth	sterile	Right thalamic abscess
Martines et al.	18	male	post-traumatic splenectomy	Dysarthria, lethargy, purulent rhinorrhea, fever	6th dental element of the left side	Bacteroides, alpha-hemolytic Streptococci	Subdural empyema secondary to sinusitis with
Andersen and Horton	70	male	Hepatitis A (1983)	Left shoulder, neck, and chest numbness; "heaviness" without pain; altered sensation in left upper chest and arm.		gram-positive anaerobic coccus and a Gram-negative anaerobic rod	BA
Wong et al.	37	male	none	Complaints of headache, visual disturbances, throbbing pain in the whole head, blurred vision, colored lights, "blotches and fuzzy spots," photophobia, phonophobia, vertigo, and chills	Left upper and lower wisdom teeth	missing	Occipital lobe abscess, later identified as esophageal squamous cell carcinoma.
Wohl et al.	44	male	migraine	Severe headache, visual disturbances, confusion, fever	Missing	Microaerophilic streptococci	Migraine complicated by vascular infarction
Nair et al.	40	male	missing	Swelling in the right temporal region for 4 months; Holocranial headache for 15 days; Sinuses over swelling with pus discharge 24h after admission	Yes	missing	Calvarial tuberculosis; Osteomyelitis of right parietal bone;
Chandy et al.	21	male	respiratory disease (sepsis and right lung empyema)	Fever, frontal headache, scalp and forehead swelling, left-sided rhinorrhea, nasal congestion.	Left maxillary molar (tooth no. 15)	Streptococcus intermedius and Bacteroides melaninogenicus infection.	Pott's abscess
Cariati et al.	46	male	missing	Temporomandibular pain, swelling, fever	Tooth 38	gram-positive Cocos	Bacterial meningitis
Reddy et al.	58	male	missing	Swelling on the left side of the face; diplopia; periorbital ecchymosis; left eye symptoms	Left maxillary posterior region	missing	Complications from Cavernous Sinus Thrombosis; Death due to CST complications
Sakashita et al.	62	male	Hypertension, Diabetes	Diplopia, pain in the back of the right eye, headache	missing	Fusobacterium sp.; Pavimonas micra	Subarachnoid and intraparenchymal abscess, lung abscess, massive intracerebral hemorrhage, fusiform aneurysms in the left middle cerebral artery, cerebral infarction, cerebral atrophy
Strojnik et al.	12	male	none	Severe right hemiparesis, more pronounced in the leg	missing	Streptococcus intermedius, Streptococcus beta-haemolyticus group F, Fusobacterium species, and gram-negative rods	BA
Clancy et al.	55	female	Chronic right-sided hearing impairment	Left retro-orbital headache, right hemisensory loss, unsteady gait	Left lower molar	Gram-positive cocci initially, later Streptococcus mitis and A. meyeri	BA
Okada et al.	58	female	Hypertension	Bleeding from red and swollen gingivae, loosening of teeth, diastema formation, extrusion, periodontal pocket formation	Upper lateral incisor	missing	Cause of death: Subarachnoid hemorrhage
Reddy et al. (b)	55	male	Diabetes Mellitus	Left-sided toothache, swelling, fever, frontal headaches	Left second upper premolar: 25	missing	left temporo-frontal hemorrhagic venous infarction
Brady et al.	68	male	none	Sudden onset slurred speech, left-sided facial droop, and left upper limb weakness. VII nerve palsy. Poor dentition. Pan-systolic murmur.	Non-traumatic loss of a tooth one week before admission	missing	BA
Clifton et al.	56	male	Hypertension, cholecystectomy, obstructive sleep apnea	Mental changes, dry cough, intermittent fever, tunnel vision, memory lapse, headache, neck and back pain, nausea, vomiting	Left the second molar	Gram-positive anaerobic streptococcal ns	BAs; Non-convulsive status epilepticus
Funakoshi et al.	57	female	Hypertension	Headache, Left arm numbness and weakness	Dental problems requiring tooth extractions	A. meyeri and Fusobacterium nucleatum	Intracranial subdural abscess
Yoshii et al.	54	male	none	Severe headache and malaise, no nausea or vomiting	Second and third molars of the left lower jaw	Peptostreptococcus tetradius, Streptococcus milleri, Streptococcus salivarius, Capnocytophaga spp.	Bacterial meningitis, later complicated by a right subdural empyema
Shibata et al. (a)	62	male	Esophageal cancer, Type 2 diabetes mellitus	Headache, fever, motor aphasia, right hemiparesis	Right maxillary second premolar and second molar	S. intermedius	BAs; patient deceased 6 months after surgery.
Shibata et al. (b)	68	male	Advanced non-small-cell lung cancer	Left hemiparesis and fever	missing	S. intermedius	BA secondary to squamous cell carcinoma and apical periodontitis after tooth extraction
Verma et al.	68	male	none	Malaise, numbness in feet, lower limb weakness, choking, respiratory distress	Right upper jaw tooth	Streptococcus intermedius infection	Medullary abscess secondary to tooth extraction,
Wu et al.	32	male	none	Progressive headache left upper limb weakness, left facial palsy	missing	sterile	OBAs with septic embolic ischemic stroke
Singh et al.	47	male	Polycystic kidney disease (PCKD), Hypertension (HTN)	Toothache, Right eye pain, Orbital swelling, Fever, Dyspnea	Tooth extraction 10 days before symptom onset	sterile	Mucormycosis, Kluyvera intermedia, Pseudomonas aeruginosa sepsis, Acute infarcts, Thrombosis, Cavernous sinus thrombosis
Naganawa et al.	76	male	Hyperthyroidism, Myocardial Infarction, Chronic Subdural Hematoma, Aortic Dissection, Chronic Kidney Disease, Hypertension, Chronic DIC	Spontaneous pain in the upper front teeth region	Maxillary left central and lateral incisors, Right central incisor	missing	Death due to Intracranial Hemorrhage associated with Aortic Dissection and DIC
Choi et al.	39	male	History of Behçet's disease, particularly NBD	Hypesthesia of the left face and extremity - Ataxia -Memory disturbance - Disorientation	Molar tooth extraction	Sterile	hemorrhagic infarction
Hibberd et al.	11	male	none	2-week history of dull continuous headache, 1-week history of nausea and vomiting	Lower left second primary molar (tooth 75)	Streptococcus anginosus (day 2)	Temporoparietal intracerebral abscess
Prabhu et al.	70	male	Uncontrolled diabetes mellitus	Altered sensorium, vomiting, decreased oral intake, right facial swelling	Right tooth	mucormycosis	neural invasion
Hobson et al.	35	female	none	Severe headache, facial swelling, mental status changes	Left maxillary third molar	Suggestive of bacterial meningitis	Acute meningoencephalitis, subdural empyema, intraparenchymal hemorrhage, neurologic deficits
Heckmann et al.	77	female	none	Neck pain, pronounced neck stiffness	Fractured first premolar in the left maxilla	Streptococcus intermedius (Milleri)	Epidural abscess
Chang et al.	6.7	male	Ebstein’s anomaly, intellectual disabilities	Sudden vomiting, loss of consciousness, facial spasm	Left lower deciduous central and lateral incisors	Streptococcus milleri group and Methicillin-resistant Staphylococcus aureus	BA
Chang et al.	63	female	none	Right frontal headaches, puffiness of the right eye, fever, diplopia, high fever, right-eye ptosis, limitation of eyeball movement, stiff neck, positive meningeal signs, muscle weakness, sensation responsive to pain stimulation, leucocytosis, neutrophilia.	Tooth extraction	Fusobacterium nucleatum	Bacterial meningitis and ischemic changes. Subsequent left-side hemiplegia.
Vargas et al.	18	male	none	Headache, vomiting, aphasia, weakness in left extremities, behavior and mood alterations, fever	Multiple teeth were extracted three months before admission	Arcanobacterium haemolyticum	BA
Ng et al.	33	male	ecstasy adiction visited prostitutes	Acute confusion; semiconscious (GCS: 10/15); urinary incontinence; fever; upper respiratory tract symptoms; expressive dysphasia; right-sided pyramidal signs; apical pansystolic murmur; no Kussmaul’s breathing; neck stiffness; no Kernig’s or Brudzinski’s signs	Yes (two weeks before admission)	sterile	Meningitis and Brain infarct; Endocarditis with mitral valve vegetation
Alfano et al.	50	female	Ketoacidotic diabetic coma	Loss of consciousness, swelling, tenderness of the right cheek	Right first premolar	Combined mucormycosis and aspergillosis	Ischemia
Corre et al.	63	female	Hereditary haemorrhagic telangiectasia (HHT)	Acute confusion, fever, and aphasia	10 teeth	Fusobacterium nucleatum and Staphylococcus epidermidis	BA
Hayashi et al.	6	female	none	Fever, severe headache, neck stiffness, nausea, vomiting	Front milk tooth (exact identity )	Group A Streptococcus (GAS)	BA
Liao et al.	44	male	missing	Progressive headache, fever >39°C, neck stiffness	Dental extraction performed 2 days before the onset of headache	P. alactolyticus and MTB	Bacterial meningitis
Lin et al.	78	male	missing	Shortness of breath and fever following tooth extraction	missing	Streptococcus anginosus	BA with intracerebral hematoma; discharged with left hemiparesis
Al Moussawi et al.	56	female	Ductal carcinoma in situ, hypothyroidism, diverticulosis	Dizziness, worsening headaches, blurry vision	missing	Streptococcus intermedius (from pus drainage)	Abscess in the right cerebellar hemisphere sigmoid diverticulitis with an adjacent abscess. Complete recovery after surgical drainage.

**OBA:**Odontogenic brain abscess ; **BA:**Brain Abscess, **MTB:**Mycobacterium tuberculosis

In the initial search, a total of 2,345 articles were identified from various databases. After removing duplicates, 1,987 articles remained. Following the screening of titles and abstracts, 1,634 articles were excluded as they did not meet the inclusion criteria. Subsequently, full-text assessment was performed on the remaining 353 articles, leading to the exclusion of an additional 311 articles.

Finally, after applying the eligibility criteria, 42 articles [23][24][25][26][27][28][29][30][31][32][33][34][35][36][37][38][39][40][41][42][43][44][45][46][47][48][49][50][51][52][53][54][55][56][57][58][59][60][61][62][63][64] were included in the systematic review for data extraction and analysis, as shown in Figure-1. The main characteristics of the cases are shown in Table-1.

Among 42 studies, 47 cases were included in the study. There were 25 cases of brain abscess. Amorim et al. describe an odontogenic brain abscess with hydrocephalus. Pallesen et al. encountered multiple brain abscesses attributed to Streptococcus intermedius and Staphylococcus warneri, leading to subsequent complications such as subdural empyema and focal epileptic seizures. Hollin et al. identified a parietal abscess, while Hollin & Gross observed a right thalamic abscess. Andersen and Horton and Strojnik et al. reported brain abscesses. Wong et al. noted an occipital lobe abscess following wisdom teeth extraction, later identified as esophageal squamous cell carcinoma. Chandy et al. reported a Pott’s abscess with Streptococcus intermedius and Bacteroides melaninogenicus infection. Sakashita et al. documented a complex case involving subarachnoid and intraparenchymal abscesses, lung abscesses, intracerebral hemorrhage, fusiform aneurysms, cerebral infarction, and cerebral atrophy. Clancy et al. reported a brain abscess secondary to Streptococcus mitis and A. meyeri infection. Brady et al. observed a brain abscess in conjunction with mitral regurgitation and left-sided weakness requiring rehabilitation. Clifton et al. documented brain abscesses along with non-convulsive status epilepticus due to hydrocephalus and hypertension. Funakoshi et al. reported an intracranial subdural abscess caused by A. meyeri and Fusobacterium nucleatum. Shibata et al. described brain abscesses secondary to chronic suppurative apical periodontitis, squamous cell carcinoma, and apical periodontitis after tooth extraction. The patient died in 6 months. Verma et al. encountered a medullary abscess secondary to tooth extraction with Streptococcus intermedius infection. Wu et al. reported odontogenic brain abscesses with septic embolic ischemic stroke. Hibberd et al. observed a temporoparietal intracerebral abscess, dental abscess, and Streptococcus anginosus infection. Heckmann et al. documented an epidural abscess secondary to dental extraction. Chang et al. identified a brain abscess caused by Streptococcus milleri group infection. Vargas et al. reported a brain abscess caused by Arcanobacterium haemolyticum. Corre et al. linked hereditary haemorrhagic telangiectasia (HHT) with neurological complications of dental extraction. Hayashi et al. documented a brain abscess with meningitis due to Group A Streptococcus (GAS) infection. Lin et al. reported a Streptococcus anginosus brain abscess with intracerebral hematoma. Al Moussawi et al. encountered an abscess in the right cerebellar hemisphere originating from Streptococcus intermedius, coupled with sigmoid diverticulitis and an adjacent abscess, ultimately achieving complete recovery after surgical drainage.

There were 13 cases of meningitis. The reported cases depict a range of meningitis presentations with diverse etiologies and complications. Hollin et al. (a) and Hollin et al. (b) documented subdural empyema with diffuse leptomeningitis, the former linked to tooth extraction complications. Hollin & Gross (a) reported subdural empyema secondary to tooth extraction, highlighting dental procedures as potential sources of intracranial infections. Martines et al. observed subdural empyema secondary to sinusitis with Bacteroides and alpha-hemolytic Streptococci infection, underscoring the significance of sinus-related complications. Nair et al. encountered calvarial tuberculosis with osteomyelitis of the right parietal bone, confirming TB infection through positive Mantoux and TB interferon gamma tests. Cariati et al. reported bacterial meningitis of dental origin, emphasizing the oral-health-related nature of the infection. Yoshii et al. documented bacterial meningitis, later complicated by a right subdural empyema. Prabhu et al. reported invasive zygomycosis (mucormycosis) with extensive angioinvasion and neural invasion, illustrating a rare but severe form of fungal meningitis. Hobson et al. observed acute meningoencephalitis with additional complications such as left pterygoid muscle abscess, subdural empyema, intraparenchymal hemorrhage, and resulting neurologic deficits. Chang et al. reported bacterial meningitis secondary to Fusobacterium nucleatum, complicated by ischemic changes in the brain and subsequent left-side hemiplegia. Ng et al. documented meningitis and septic emboli, brain infarction, and endocarditis with mitral valve vegetation, showcasing the systemic impact of the infection. Alfano et al. described combined mucormycosis and aspergillosis of the rhinocerebral region, emphasizing the potential for multiple fungal infections. Liao et al. reported bacterial meningitis with coinfection of P. alactolyticus and Mycobacterium tuberculosis (TB), highlighting the coexistence of different pathogens in meningitis cases. These cases collectively underscore the diverse etiologies, complications, and severity associated with meningitis, emphasizing the importance of prompt diagnosis and appropriate management.

The cases reported 8 cerebrovascular accidents (CVAs) with distinct etiologies and complications. Kroppenstedt et al. documented a pituitary macroadenoma with a secondary infection post-tooth extraction, emphasizing the potential complications associated with dental procedures. Calderon-Miranda observed a subdural hematoma, highlighting the intracranial consequences of traumatic injuries. Wohl et al. reported migraine complicated by vascular infarction, showcasing the association between migraines and cerebrovascular events. Reddy et al. reported complications from cavernous sinus thrombosis (CST) leading to death, underscoring the severity of this condition. Okada et al. identified subarachnoid hemorrhage as the cause of death, indicating a rupture of blood vessels into the space surrounding the brain. Reddy et al. (b) documented a rhino-orbital infection from a dental source with cavernous sinus extension causing left temporo-frontal hemorrhagic venous infarction, illustrating the potential for localized infections to impact venous structures. Singh et al. encountered a complex case involving mucormycosis, Kluyvera intermedia, Pseudomonas aeruginosa sepsis, acute infarcts, thrombosis, and cavernous sinus thrombosis, highlighting the multifactorial nature of cerebrovascular complications. Naganawa et al. reported death due to intracranial hemorrhage associated with aortic dissection and disseminated intravascular coagulation (DIC), emphasizing the systemic impact of vascular disorders. Choi et al. documented Behçet’s disease, particularly neuro-Behçet’s disease (NBD), illustrating the association between inflammatory conditions and cerebrovascular involvement.

### Descriptive statistics

In this study involving 47 participants (34 males, 13 females), the mean age for male participants was 45.17 ± 20.60 years, while the mean age for female participants was 49.54 ± 19.63 years. The independent t-test revealed no statistically significant difference in mean age between genders (P = 0.5134).

The most common symptom overall was headache, reported by 55.32% of all participants. Fever was presented in 38.3% of the cases. Laterality symptoms, such as weakness, hemiparesis, and sensory disturbances, were noted in 25.53% of individuals. Neck pain or neck rigidity followed, with a prevalence of 14.89%, while symptoms encompassing dizziness, fatigue, malaise, and vertigo were observed in 19.15% of cases. Nausea and vomiting were reported in 14.89% of cases. When examining gender differences, the prevalence of neck pain/neck rigidity was significantly higher in females (30.77%) compared to males (8.82%), with a P-value of 0.042. Nausea and vomiting also showed a notable gender difference, with 30.77% of females experiencing these symptoms compared to 8.82% of males (P = 0.028). While fever was a prevalent symptom in both genders, there was no significant difference observed (P = 0.632).

Other rare symptoms are as follows: gait disturbances, facial droop/spasm, cardiac symptoms, focal convulsions, rhinorrhea/sinusitis, insomnia, personality changes, and blood pressure variations.

Among males, 29.41% had no underlying diseases, while 38.46% of females fell into the same category, resulting in an overall rate of 31.91%. Hypertension was reported in 11.76% of males and 15.38% of females, with a combined prevalence of 17.65%. Diabetes was less prevalent, with 5.88% of males and no cases reported among females. Among the patients, the distribution of various disorders was as follows: hypothyroidism was reported in 2 individuals, and post-traumatic splenectomy and hepatitis A conditions were each identified in 1 patient. Additionally, respiratory diseases were observed in 3 cases. Cancers, kidney diseases, and congenital disorders each affected 2 patients. Cardiac diseases, stroke/cerebral hemorrhages, neurological diseases, addiction, and surgical diseases were each reported in 1 patient.

The results of various cultures from different studies revealed a diverse spectrum of microbial isolates. Streptococcus intermedius was identified in multiple cases, either alone or in combination with other pathogens such as Staphylococcus warneri, Streptococcus beta-haemolyticus group F, and Fusobacterium species. Staphylococcus CJWXU.S and nonhemolytic streptococci were observed in separate cases. Gram-positive anaerobic cocci and Gram-negative anaerobic rods were detected together in one case. Microaerophilic streptococci were reported, as well as Gram-positive cocci initially, later identified as Streptococcus mitis and A. meyeri. Peptostreptococcus tetradius, Streptococcus milleri, Streptococcus salivarius, and Capnocytophaga spp. were identified together in a distinct case. Other findings included Fusobacterium nucleatum, Arcanobacterium haemolyticum, and Methicillin-resistant Staphylococcus aureus. Notably, sterile cultures were reported in several cases, while Streptococcus anginosus was specifically mentioned in one case, suggesting a potential association with bacterial meningitis.

Time from tooth extraction to emergency room visit was examined for 40 observations, indicating a mean duration of 19.73 days, with a standard deviation of 31.01 days. The range spanned from a minimum of 2 days to a maximum of 180 days.

In cases where data is available, 29.79% (14 cases) of dental extractions are from the upper jaw, 12.77% (6 cases) to the lower jaw, and 4.26% (2 cases) involve both the upper and lower jaws simultaneously.

The regression analysis conducted on death among cases of neurosurgical complications after tooth extraction reveals that age and gender do not significantly influence the likelihood of death, as indicated by odds ratios of 1.07 (95% CI: 0.98-1.17) and 0.86 (95% CI: 0.08-9.11), respectively, with P-values of 0.108 and 0.901. However, a statistically significant association is observed between the number of preexisting diseases and death, with an odds ratio of 2.15 (95% CI: 1.08-4.29) and a P-value of 0.03, suggesting that each additional preexisting disease increases the odds of death by approximately 2.15 times. Conversely, the time from tooth extraction to ER symptoms does not significantly impact the likelihood of death, with an odds ratio of 1.01 (95% CI: 0.98-1.04) and a P-value of 0.718. hypertension shows a statistically significant association with death, as evidenced by an odds ratio of 9.75 (95% CI: 1.07-89.2) and a P-value of 0.044. This suggests that individuals with hypertension are at significantly higher odds of death compared to those without hypertension.

The quality of included studies was assigned as 16 studies with high quality, 15 with intermediate, and 16 studies with low quality, as shown in Table-4.

## Discussion

**Table:** Table2. Symptoms comparison among genders

	male	female	Total	P-value
n	34	13	47	-
Headache	18(52.94%)	8(61.54%)	26(55.32%)	0.41
Neck pain/neck rigidity	3(8.82%)	4(30.77%)	7(14.89%)	0.042
Dizziness/Fatigue/malaise/vertigo	8(23.53%)	1(7.69%)	9(19.15%)	0.254
Nausea & vomiting	3(8.82%)	4(30.77%)	7(14.89%)	0.028
Fever	14(41.18%)	4(30.77%)	18(38.3%)	0.632
Laterality (weakness, hemiparesis, sensory)	11(32.35%)	1(7.69%)	12(25.53%)	0.103
Chest pain	1(2.94%)	0(0%)	1(2.13%)	0.548
Difficulty in speaking/slurred speech/Dysarthria/aphasia	4(11.76%)	2(15.38%)	6(12.77%)	0.665
Numbness in any part of the body	5(14.71%)	0(0%)	5(10.64%)	0.159
Cough/respiratory	4(11.76%)	0(0%)	4(8.51%)	0.214
Memory disturbances	4(11.76%)	0(0%)	4(8.51%)	0.214
Mental state changes (confusion)	10(29.41%)	2(15.38%)	12(25.53%)	0.387
Visual disturbances	7(20.59%)	0(0%)	7(14.89%)	0.088

**Table:** Table3. Logistic regression of the predictors of death among patients with neurosurgical complications after tooth extraction

	OR	lower 95%CI	upper 95%CI	P-value
age	1.07	0.98	1.17	0.108
gender	0.86	0.08	9.11	0.901
number of preexisting diseases	2.15	1.08	4.29	0.03
time from tooth extraction to ER symptoms	1.01	0.98	1.04	0.718
hypertension	9.75	1.07	89.2	0.044

Our study aimed to investigate the characteristics and outcomes of patients experiencing neurosurgical complications following tooth extraction. This study demonstrates typical cases of post-tooth extraction neurosurgical complications. While being indicated, we cannot delay dental care in any case, there should be caution regarding male patients who have more than one underlying disease and need upper jaw dental manipulation, while female and lower jaw incidents are also possible. Risk factors of post-dental extraction short-term complications might include traumatic extraction, tobacco use, oral contraceptives, female gender, and preexisting infections [61][62][63]. Additionally, hemorrhage after extraction could be linked to the expertise level of practitioners and patient-specific factors like bleeding disorders [62][63][64]. Poor oral hygiene, smoking, and underlying systemic conditions are associated with suppurative alveolitis, while post-extraction pain may result from factors like extraction complexity, insufficient pain control, and individual pain tolerance [62][63][64]. Moreover, postoperative infections are more prevalent in individuals with compromised immune systems and inadequate oral hygiene practices [61][62][63][64]. But, in our study, male gender was more prominent. However, the incident of post-dental extraction neurosurgical complication is not a short-term outcome and mostly happened after 2 weeks of the dental extraction. So, it seems that the risk factors and pathophysiological nature of these complications are far away from the classic complications of dental extraction.

In our study, hypertension and multiple preexisting conditions were significant predictors of death. The relationship between hypertension and neurosurgical complications can be complex. There is a case report of the sudden increase in blood pressure and intracerebral hemorrhage in a normotensive patient and death in the dentistry room [65]. Research suggests a link between trigeminal nerve stimulation, including methods like trigeminal nerve combing or proprioceptive stimulation, and alterations in arterial blood pressure [66][67]. The involvement of trigeminal nerve inputs in governing cerebral blood flow also suggests potential implications for blood pressure regulation [68]. It’s generally advised to avoid emergency dental procedures in patients with severely elevated blood pressure (>180/110 mmHg) due to increased risks [69].

Most vases were gram-positive Cocos bacteria (22 cases, 46.8%) as the source of infection and abscess, but rare cases of Capnocytophaga spp. (Flavobacteriia), Peptostreptococcus tetradius (Clostridia) in the study of Yoshii et al. [40], MTB and aspergillosis in Liao et al. study [58], and Arcanobacterium haemolyticum in Vargas et al. study [52] were seen. Most observed co-infection was a coincidence of isolates of gram-negative anaerobic rods and gram-positive Bacilli in 5 cases. Gram-positive cocci bacteria, like Streptococcus intermedius and Anaerococcus prevotii, have been associated with brain abscesses stemming from dental origins [70]. These microbes reside naturally in the oral cavity and can lead to infections if introduced into the bloodstream, frequently due to dental procedures or infections [71]. Anaerococcus prevotii, characterized as a gram-positive coccus, has emerged as a potential pathogen responsible for brain abscesses, some of which are linked to dental sources [72]. Similarly, Parvimonas micra, another gram-positive anaerobic coccus prevalent in the oral mucosa, has been linked to cerebral abscesses, often arising from dental infections [73].

Maurer et al. reported a case of meningitis that didn’t require neurosurgical intervention and CSF culture was sterile [74]. In our study there were 7 cases in which no infective source was isolated from abscess or CSF cultures. A seldom-seen event, meningitis caused by Capnocytophaga spp. is infrequent but warrants consideration in individuals with underlying health issues or predisposing factors, like compromised immune systems [75]. Also, it was reported after a dog bite [76]. Another study showed that lower jaw wisdom tooth extraction causes more cases of infection than upper jaw [77]. However, in our study, most cases were experiencing the incident after the upper jaw manipulation. The reason behind this difference could be attributed to the diverse pathophysiology of the complications. However, it’s necessary to acknowledge the limitations of this study. Firstly, the relatively small sample size of cases limits the generalizability of the findings. Also, we cannot estimate the prevalence of this condition in public as no data is available from any cohort and the mentioned risk factors are in fact the classic representation of post-tooth extraction neurosurgical complications. Furthermore, the lack of a control group hinders the ability to establish causality or determine the true prevalence of complications following tooth extraction.

## Conclusion

In conclusion, this systematic review and individual patient meta-analysis showed characteristics and outcomes of neurosurgical complications following tooth extraction. The study revealed a variety of complications including brain abscess, meningitis, cerebrovascular accidents, and others, with notable gender differences in symptom presentation. Headache and fever emerged as the most common symptoms, showing their importance in prompt evaluation and management, particularly in male patients with pre-existing conditions undergoing upper jaw teeth extraction.

## Conflict of Interest

The authors have no conflicts of interest relevant to this article to disclose.
